# Type I-F CRISPR-Cas Distribution and Array Dynamics in *Legionella pneumophila*

**DOI:** 10.1534/g3.119.400813

**Published:** 2020-01-14

**Authors:** Shayna R. Deecker, Alexander W. Ensminger

**Affiliations:** * Department of Biochemistry and; † Department of Molecular Genetics, University of Toronto, Ontario, Canada

**Keywords:** bacteriology, CRISPR-Cas, Legionella, microbiology

## Abstract

In bacteria and archaea, several distinct types of CRISPR-Cas systems provide adaptive immunity through broadly similar mechanisms: short nucleic acid sequences derived from foreign DNA, known as spacers, engage in complementary base pairing with invasive genetic elements setting the stage for nucleases to degrade the target DNA. A hallmark of type I CRISPR-Cas systems is their ability to acquire spacers in response to both new and previously encountered invaders (naïve and primed acquisition, respectively). Our phylogenetic analyses of 43 *L. pneumophila* type I-F CRISPR-Cas systems and their resident genomes suggest that many of these systems have been horizontally acquired. These systems are frequently encoded on plasmids and can co-occur with nearly identical chromosomal loci. We show that two such co-occurring systems are highly protective and undergo efficient primed acquisition in the lab. Furthermore, we observe that targeting by one system’s array can prime spacer acquisition in the other. Lastly, we provide experimental and genomic evidence for a model in which primed acquisition can efficiently replenish a depleted type I CRISPR array following a mass spacer deletion event.

Microorganisms have evolved over millions of years to survive in harsh environments, backed in part by immune strategies that protect against foreign genetic elements, such as viral phages and foreign DNA elements (van Houte *et al.* 2016). Clustered regularly interspaced short palindromic repeats (CRISPR) coupled with associated *cas* genes form a potent adaptive immune response in numerous prokaryotic species (Barrangou *et al.* 2007; Brouns *et al.* 2008; Marraffini and Sontheimer 2008). These systems have been classified into six major types, which are further divided into various subtypes, based on their mechanism of action and Cas protein content (Makarova *et al.* 2011; 2015; Koonin *et al.* 2017).

A CRISPR-Cas response to invading DNA occurs in three distinct phases: adaptation, expression and interference (Barrangou *et al.* 2007; Brouns *et al.* 2008; Marraffini and Sontheimer 2008). In the adaptation phase, the CRISPR-Cas system acquires a DNA sequence (spacer) from the invader and integrates it into an array of spacers interspersed with repetitive sequences (Ishino *et al.* 1987; Mojica *et al.* 2000; Jansen *et al*. 2002b; Barrangou *et al.* 2007; Jackson *et al.* 2017). The spacers can be derived from foreign elements whose infection was unsuccessful, such as defunct phage (Hynes *et al.* 2014), from free DNA ends generated during double strand breaks (Levy *et al.* 2015; Modell *et al.* 2017) or following phage DNA injection into the host cell (Modell *et al.* 2017). Once acquired, these spacers form the basis of immunological memory for the bacterium (Barrangou *et al.* 2007; Jackson *et al.* 2017). During the expression phase, the array is transcribed and processed to form CRISPR RNA (crRNA) molecules which form a surveillance complex with Cas proteins (Brouns *et al.* 2008; Haurwitz *et al.* 2010). Infection by an invading genetic element initiates the interference step, wherein the surveillance complex recognizes and binds the foreign DNA via base-pairing with the complementary crRNA and cleaves it, effectively neutralizing the threat to the host (Brouns *et al.* 2008; Jore *et al.* 2011; Wiedenheft *et al.* 2011; Westra *et al.* 2012).

Despite the sophistication of CRISPR-Cas systems, phages and other foreign DNA elements can still escape CRISPR-Cas targeting (Samson *et al.* 2013; Stanley and Maxwell 2018). A common mechanism of escape is the accumulation of random mutations within the foreign element that either prevent complementary base pairing with crRNAs during interference or abolish target recognition (*i.e.*, through protospacer adjacent motif [PAM] mutations) (Deveau *et al.* 2008; Semenova *et al.* 2011; Datsenko *et al.* 2012). CRISPR-Cas systems can overcome this escape by acquiring new spacers; in fact, imperfect CRISPR targeting often leads to a highly efficient “primed” acquisition response, providing an intrinsic mechanism to protect against mutational escape (Swarts *et al.* 2012; Datsenko *et al.* 2012; Savitskaya *et al.* 2013; Fineran *et al.* 2014; Richter *et al.* 2014). Primed acquisition has been studied in type I-B (Li *et al.* 2014a; 2014b; 2017), I-C (Rao *et al.* 2017), I-E (Swarts *et al.* 2012; Datsenko *et al.* 2012; Savitskaya *et al.* 2013; Fineran *et al.* 2014; Xue *et al.* 2015; Jackson *et al.* 2019) and I-F (Richter *et al.* 2014; Vorontsova *et al.* 2015; Staals *et al.* 2016; Heussler *et al.* 2016; Jackson *et al.* 2019) CRISPR-Cas systems. Interference-driven acquisition, or targeted acquisition, has also been observed in type I-C (Rao *et al.* 2017), type I-E (Kuznedelov *et al.* 2016) and I-F systems (Staals *et al.* 2016), wherein a primed acquisition response occurs against a target with a perfect match to a spacer already within the array.

*Legionella pneumophila* is a Gram-negative bacterium and the causative agent of Legionnaires’ disease (Brenner *et al.* 1979). Most isolates possess any of three different CRISPR-Cas systems: type I-C, I-F and/or II-B (D’Auria *et al.* 2010; Rao *et al.* 2016). Our lab has recently shown that all three types of CRISPR-Cas systems found in *L. pneumophila* isolates are active (Rao *et al.* 2016) and we have characterized the targeted acquisition response for the type I-C system (Rao *et al.* 2017). While much of this work to date has focused on the I-C systems of *L. pneumophila*, the type I-F systems of this pathogen are highly protective, remarkably diverse with respect to spacer content, and are frequently found on plasmids – suggesting that they may be circulated via horizontal gene transfer (Rao *et al.* 2016). In this study, we perform the first comprehensive phylogenetic analysis of the *L. pneumophila* type I-F systems and test a model by which horizontal acquisition of a mobile type I-F CRISPR-Cas system could replenish a collapsed chromosomal array.

## Materials and Methods

### Bioinformatic analyses

Bioinformatic analyses of the Illumina sequence data were performed as described previously (Rao *et al.* 2017). Briefly, the raw paired-end reads were merged using FLASH (Magoc and Salzberg 2011), and any unpaired reads were subsequently quality trimmed using Trimmomatic (Bolger *et al.* 2014). These processed reads were then combined and analyzed using a Perl script (available upon request) that annotated existing spacers (S), newly acquired spacers (X), repetitive sequences (R) and the downstream sequence (D). The newly acquired spacers were aligned to the priming plasmid, the *L. pneumophila* str. Lens chromosome or the *L. pneumophila* str. Lens plasmid using BLASTN (Altschul *et al.* 1990). The results from the BLASTN alignment for the priming plasmid were then processed to obtain coverage per nucleotide, and plotted on the reference sequence using Circos (Krzywinski *et al.* 2009).

For the bioinformatic analyses of the *L. pneumophila* type I-F system diversity, *L. pneumophila* draft genomes and completed genomes were downloaded from the European Nucleotide Archive and NCBI respectively (Table S1). Type I-F CRISPR-Cas systems were identified using CRISPRCasFinder (Couvin *et al.* 2018) and CRISPRDetect (Biswas *et al.* 2016). All genomes with type I-F systems present were annotated using Prokka (Seemann 2014). For the core genome phylogeny, pan-genome analysis was performed with Roary using the default settings and the MAFFT aligner (Page *et al.* 2015). The *cas1* genes were extracted from each CRISPR-Cas system and aligned with MUSCLE (Edgar 2004). The *cas1* gene alignment and the core genome alignment were used to create phylogenies with RAxML using the rapid bootstrapping and search for best maximum likelihood tree algorithm with 1000 bootstrap iterations (Stamatakis 2014). The RAxML trees were condensed with MEGA7 using a bootstrap support cut-off of 50% (Kumar *et al.* 2016). For the *cas1* tree, each phylogroup possesses 100% nucleotide identity in the *cas1* sequence. CRISPR array alignments, clustering and visualization were performed with CRISPRStudio (Dion *et al.* 2018). Four isolates possessed split CRISPR-Cas arrays (found at the end of separate contigs) in the draft genomes, so they were excluded from both phylogenetic analysis and CRISPRStudio analysis to minimize potential problems with array assembly (Table S1).

### Bacterial strains, plasmids and oligos used

The bacterial strains and plasmids used in this study are listed in supplementary table 2, and the oligos used in this study are listed in supplementary table 3.

The priming plasmids were created by annealing oligos (see Table S3) to create the protospacer insert with the canonical GG PAM (Mojica *et al.* 2009; Cady *et al.* 2012; Richter *et al.* 2014; Vorontsova *et al.* 2015; Staals *et al.* 2016) and subsequently ligating the insert into an ApaI/PstI-digested pMMB207 vector (Solomon *et al.* 2000). The scrambled control plasmid was created in the same manner, except it contained a 32-nt scrambled sequence in place of a targeted protospacer sequence.

Lens (chromosome) array deletion mutants were generated through allelic replacement. Briefly, 1 kb of DNA upstream and 2 kb downstream of the CRISPR array were amplified by PCR and stitched together to create an insert where the entire array, save for the last repeat, was deleted. The insert was ligated into a pJB4648 plasmid. Overnight cultures of *L. pneumophila* str. Lens were grown in ACES-buffered yeast extract (AYE) medium to an OD_600_ of ∼4.0 using two-day patches that were grown on charcoal-buffered ACES yeast extract (CYE) plates. Pellets from 4.0 ODU of culture underwent three washing steps: twice with 1 mL of ice-cold ultrapure water and once with 1 mL of ice-cold 10% glycerol. The pellet was then re-suspended in 200 μL of ice-cold 10% glycerol and 400 ng of plasmid was added to the sample. The solution was transferred to an ice-cold electroporation cuvette with a 2 mm gap and electroporated with the following settings: 2500 kV, 600 Ω and 25 mF. After electroporation, 800 μL of AYE medium was added to each sample and the samples recovered for 3 hr at 37° at 600 RPM in a shaking incubator. The samples were plated on CYE plates supplemented with 15 μg mL^-1^ of gentamycin and incubated at 37° for 3 days. Surviving colonies were patched onto CYE + gentamycin plates and grown at 37° for 2 days. Patches were subsequently struck onto CYE plates supplemented with sucrose and incubated at 37° for 3 days. Surviving colonies were patched onto CYE + sucrose plates, grown at 37° for 2 days and screened by PCR to confirm the deletion. Two independent clones were Illumina sequenced and used for subsequent replenishment assays.

### Transformation efficiency assay and population pool generation

The transformation efficiency assay was performed as we have described previously (Rao *et al.* 2016) with some modifications. Briefly, *L. pneumophila* str. Lens was electroporated as described above. The samples were plated in a dilution series on CYE plates supplemented with 5 μg mL^-1^ of chloramphenicol and incubated at 37° for 3 days. The relative transformation efficiency for each targeted plasmid was calculated as a percentage of the transformation efficiency obtained from the scrambled control plasmid. Three biological replicates were performed for each transformation efficiency assay.

Population pools for spacer acquisition experiments were generated by mixing together ≥ 50 colonies per population from a newly transformed wild-type strain on CYE plates supplemented with 5 μg mL^-1^ of chloramphenicol using AYE medium supplemented with 5 μg mL^-1^ of chloramphenicol. Population pools were made in triplicate for each transformed plasmid.

### Serial passaging on an automated liquid handler

The serial passaging of transformed *L. pneumophila* str. Lens populations was performed as described previously (Rao *et al.* 2016). Briefly, overnight cultures of the population pools in AYE medium supplemented with 5 μg mL^-1^ of chloramphenicol for plasmid maintenance were grown to an OD_600_ of ∼2.0. The culture was then back diluted to an OD_600_ of ∼0.0625 and grown in a flat-bottom 48-well plate (Greiner) in a shaking incubator at 37°. A Freedom Evo 100 liquid handler (Tecan) connected to an Infinite M200 Pro plate reader (Tecan) measured the optical density of the plate every 20 min, until an OD_600_ of ∼2.0 was reached. The cultures were then automatically back diluted to an OD_600_ of ∼0.0625 in the adjacent well to continue growth, and the remaining culture was transferred to a 48-well plate that was kept at 4°. In this manner, each saved culture represented ∼5 generations of growth. The passaging was done without selection in AYE medium to allow for plasmid loss during passaging.

### Genomic DNA extraction, PCR and agarose gel screening

Genomic DNA was extracted from the passaged cultures and the parental chromosome Lens array deletion strains using a Machery-Nagel Nucleospin Tissue kit according to the manufacturer’s protocol. The extracted samples from passaged cultures were used as a template in a 30-cycle PCR reaction with PaCeR HP Polymerase (GeneBio Systems) to amplify the leader end of the CRISPR array using primers listed in Table S3. The PCR products were then separated on a 3% agarose gel to determine if spacer acquisition (or spacer loss) had occurred based on the presence of an upper (or lower) band relative to the control sample.

### Nextera library prep and Illumina sequencing

The extracted genomic DNA from passaged cultures was prepared for leader-end array sequencing by performing a 20-cycle PCR using Kapa HiFi Polymerase (Kapa Biosystems) and the primers listed in Table S3. The PCR products were purified using a Machery-Nagel Nucleospin Gel and PCR Clean-up kit as per the manufacturer’s instructions and normalized to 1 ng using the Invitrogen Quant-iT PicoGreen dsDNA assay. The DNA was then tagmented using a Nextera XT tagmentation kit as per the manufacturer’s instructions. The tagmented products were sequenced with a paired-end (2 × 150 bp) sequencing run on an Illumina NextSeq platform at the Centre for the Analysis of Genome Evolution and Function (CAGEF) at the University of Toronto.

The genomic DNA from the parental chromosome Lens array deletion strains was normalized to 1 ng using PicoGreen. The DNA was then tagmented using a Nextera XT tagmentation kit as per the manufacturer’s instructions. The tagmented products were sequenced with paired-end (2 × 150 bp) sequencing in-house on an Illumina MiniSeq platform.

### Data availability

Strains and plasmids are available upon request. Raw Illumina reads have been deposited into the NCBI sequence read archive under the BioProject PRJNA433194. Supplemental material available at figshare: https://doi.org/10.25387/g3.11367821.

## Results

### Phylogenetic analyses suggest widespread horizontal exchange of L. pneumophila type I-F systems

**Figure 1:**
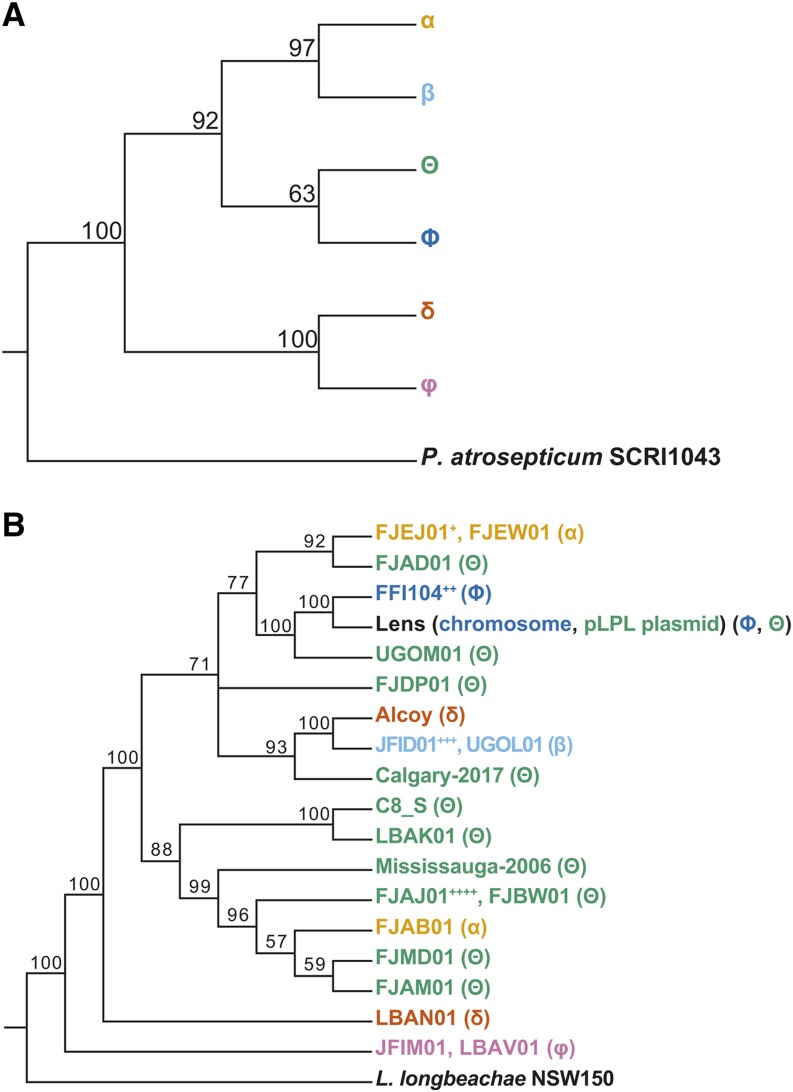
Phylogenetic analysis of type I-F CRISPR-Cas system diversity in *L. pneumophila* reveals a horizontal distribution across isolates. *L. pneumophila* draft and completed genomes were analyzed using CRISPRDetect and CRISPRCasFinder to identify type I-F systems present within the genomes. Isolates that possessed type I-F systems were subjected to phylogenetic analyses of their *cas1* gene and core genome. The core genome alignment for the examined isolates was determined by Roary. Isolate names are color-coded based on the *cas1* gene phylogeny to allow for comparison between the analyses. The isolates used in this analysis and their accession numbers can be found in supplementary table 1. A) The *cas1* gene phylogeny for *L. pneumophila* isolates with type I-F systems reveals six different *cas1* groups. B) The core genome phylogeny of the examined isolates is not congruent with the *cas1* phylogeny, suggesting that many of the type I-F systems were horizontally acquired rather than vertically inherited. Note that Lens possesses two type I-F CRISPR-Cas systems, one on a plasmid (group Θ) and one on its chromosome (group Φ). Also note that isolates with 100% nucleotide identity in their core genome, as well as a shared *cas* gene group and redundant CRISPR array, have been collapsed to one representative in the phylogeny. Isolates with unique CRISPR arrays but shared core genomes are listed separately. Bootstrap support values for each node are indicated in both trees. Collapsed isolates include: +: FJEK01, FJEL01, FJEM01, FJEN01, FJEO01, FJEP01, FJEQ01, FJER01, FJES01, FJET01, FJEU01, FJEV01, FJEX01, FJEY01, FJEZ01, FJFA01; ++: FFI105, FFI337; +++: LAXR01; and ++++: FJBI01, FJBU01.

In order to explore the hypothesis that plasmid-based type I-F CRISPR-Cas systems in *L. pneumophila* could be circulated via horizontal gene transfer, we bioinformatically examined the diversity of type I-F CRISPR-Cas systems within this species. Using both CRISPRCasFinder (Couvin *et al.* 2018) and CRISPRDetect (Biswas *et al.* 2016), we surveyed 525 draft and 6 completed *L. pneumophila* genomes. In total, we identified 47 *L. pneumophila* isolates that possessed type I-F systems (Table S1), including 5 that we had described previously (Rao *et al.* 2016). Four of these isolates were subsequently excluded from further analyses to minimize potential problems with array assembly (Table S1). We next performed two types of phylogenetic analysis: *cas1* phylogeny (Figure 1A), which placed each CRISPR-Cas system into one of six phylotypes; and core-genome phylogeny (Figure 1B), which reflects the overall relatedness between each of the 43 isolates. A comparison of the two trees indicates a clear phylogenetic incongruence suggesting that horizontal acquisition has impacted the distribution of type I-F CRISPR-Cas systems within the species. Additionally, three of the type I-F systems were present on annotated plasmids (str. Lens pLPL, str. Mississauga-2006 and str. C8_S), with two type I-F CRISPR-Cas systems occurring in the same isolate (str. Lens). There were also two isolates that possessed both a type I-C and a type I-F CRISPR-Cas system (str. Mississauga-2006 and str. FJAD01).

**Figure 2:**
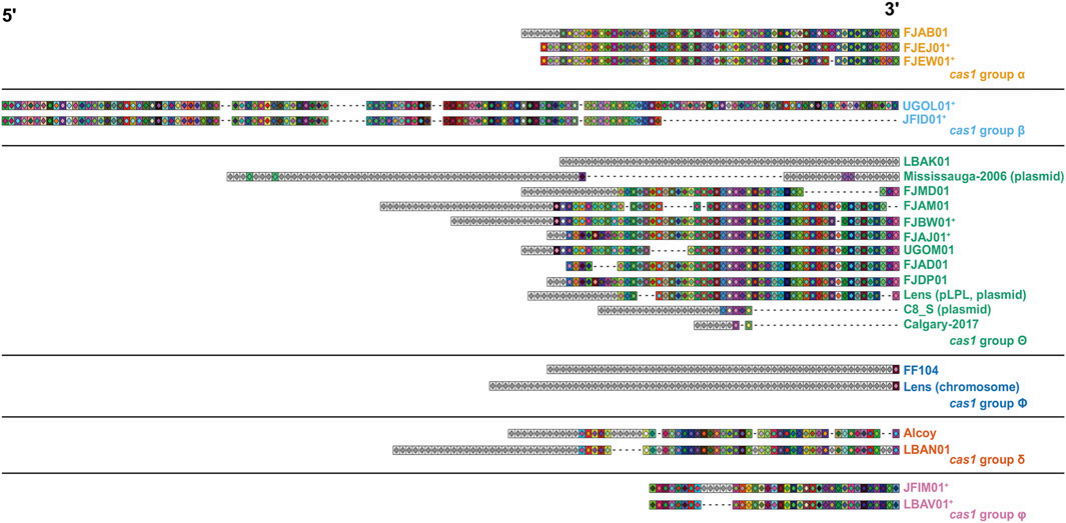
CRISPR array analysis suggests that spacer acquisition and spacer loss has contributed to array diversification in *L. pneumophila* type I-F systems. *L. pneumophila* isolates were subjected to a CRISPRStudio analysis to look at the spacer composition of their CRISPR arrays. Gray boxes denote unique spacers, colored boxes denote shared spacers and a dashed line denotes spacer loss. The isolate color coding scheme is based on the *cas1* grouping from Figure 1. Isolates denoted with a “+” within the same *cas* gene group have 100% nucleotide identity in their core genomes. Three strains have systems that reside on contigs previously annotated as plasmids (str. Lens, str. Mississauga-2006, and str. C8_S). Notably, an additional six strains (FJAD01, FJAJ01, FJAM01, FJBU01, FJBW01 and FJMD01) have systems that reside on contigs with overlapping sequence (78-89 nt) on either end, suggesting that they may also reside on a plasmid or other mobile element.

Given the results of these phylogenetic analyses, we next examined the spacer distribution across each of the arrays to determine the level of array diversification within each of the six *L. pneumophila* type I-F *cas1* phylotypes. We aligned, clustered, and visualized each of the 23 distinct *L. pneumophila* type I-F CRISPR arrays using CRISPRStudio (Dion *et al.* 2018) (Figure 2). These analyses revealed patterns consistent with both spacer acquisition and spacer loss, suggesting that both processes contribute to *L. pneumophila* type I-F CRISPR array diversity.

### The plasmid and chromosomal Lens CRISPR-Cas systems are active and adaptive

**Figure 3:**
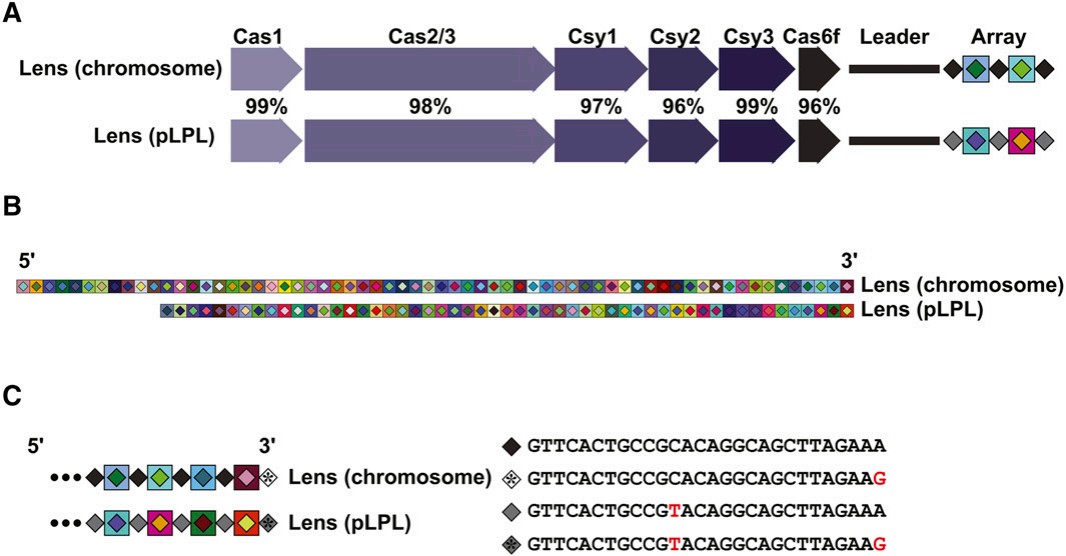
A comparison of the Lens chromosome and pLPL CRISPR-Cas systems. A) The overall pairwise amino acid identity across the Cas proteins of the two systems is approximately 97%, with individual pairwise Cas protein identities ranging from 96 to 99%. B) A CRISPRStudio alignment of the arrays for two systems shows completely unique spacer content and a differing number of spacers. C) Analysis of the repeat sequences shows that the Lens pLPL and chromosome systems have one SNP between their consensus repeats, in addition to possessing a mutated last repeat in their arrays. Mutations are denoted in red.

Given the evidence for spacer gain and loss that we observe in *L. pneumophila* type I-F arrays (Figure 2), we next decided to examine array dynamics experimentally. We focused on *L. pneumophila* str. Lens, which possesses two type I-F CRISPR-Cas systems: one on its chromosome and one on an endogenous 60 kb plasmid, pLPL (D’Auria *et al.* 2010; Gomez-Valero *et al.* 2011; Rao *et al.* 2016). The two systems have a 97.6% Cas protein identity and the repeat units between the spacers in the CRISPR array differ by only a single nucleotide (Rao *et al.* 2016) (Figure 3). The CRISPR arrays themselves are of different lengths (64 spacers for the chromosomal system and 53 for that contained on the pLPL plasmid). Despite a high degree of identity between the Cas proteins, each array contains a completely unique set of spacers (D’Auria *et al.* 2010; Rao *et al.* 2016). The presence of two remarkably similar I-F systems in *L. pneumophila* str. Lens provided us with an opportunity to examine targeted spacer acquisition in both of these largely uncharacterized CRISPR-Cas systems and the interplay between them.

**Figure 4:**
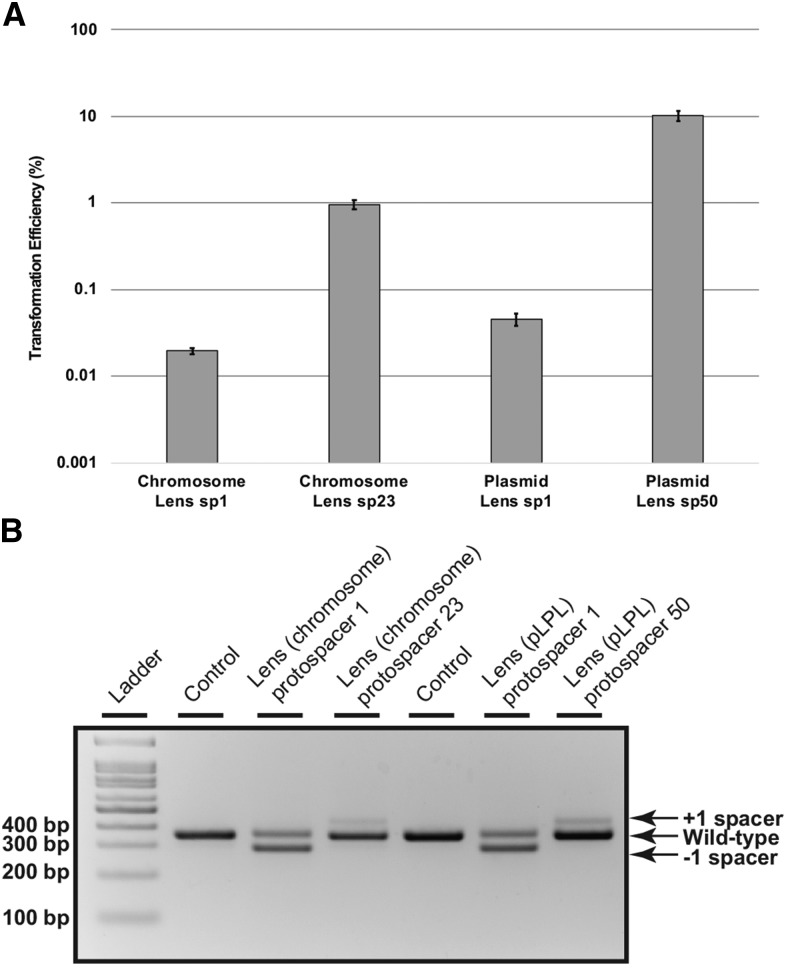
The *L. pneumophila* str. Lens chromosome and pLPL type I-F CRISPR-Cas systems are active against plasmids containing protospacers. A) *L. pneumophila* str. Lens was transformed with plasmids containing targeted protospacer sequences matched to the first spacer (sp1) or a downstream spacer (spacer 23 or spacer 50, respectively) of the Lens (chromosome) CRISPR-Cas system or the Lens (pLPL) CRISPR-Cas system. After plating on selective media and incubating for three days, transformation efficiencies were calculated as a percentage of the transformation efficiency of a control plasmid with a scrambled targeted sequence. The average for three biological replicates is shown where the error bars represent the standard error of the mean. B) Spacer acquisition and loss were analyzed using a PCR-based screen in which the leader-end of the CRISPR array for both the control samples and the transformed samples was amplified with system-specific primers to differentiate between the chromosomal Lens and the plasmid Lens arrays and visualized on an agarose gel. Products from the transformed samples were compared to the control, which contained untransformed genomic DNA. Bands representing spacer acquisition and loss are indicated.

To assess CRISPR-Cas activity in both Lens type I-F systems, we performed an established transformation efficiency assay (Marraffini and Sontheimer 2008) using two different targeted protospacer sequences: one matching the most recently acquired spacer and one matching a spacer further downstream in the array (chromosomal spacer 23 and pLPL spacer 50). Consistent with active CRISPR-Cas protection, each of the protospacer-containing plasmids exhibited reductions in transformation efficiencies relative to a scrambled protospacer control (Figure 4A). These relative transformation efficiencies ranged from 1 × 10^−2^ to 1 × 10^−4^, with the most recently acquired spacers providing ∼100-fold greater protection than spacers located further downstream in each array.

To determine whether spacer acquisition occurs within the context of a perfectly matched protospacer target - as we previously observed for a relatively permissive type I-C CRISPR-Cas system (Rao *et al.* 2016) - we pooled the transformed populations, passaged them on an automated liquid handler for 20 generations without selection, extracted their genomic DNA, and screened the leader end of the CRISPR array by PCR and agarose gel electrophoresis. While the populations transformed with plasmids encoding either protospacer 23 (chromosome) or protospacer 50 (pLPL plasmid) exhibited spacer acquisition in both Lens systems (Figure 4B), the populations transformed with protospacer 1 plasmids exhibited spacer loss, with spacer acquisition undetectable on a gel (Figure 4B). This is consistent with the level of protection we see against these plasmids (Figure 4A): in addition to spacer loss, we may have also selected for host *cas* or plasmid protospacer mutants that could preclude spacer acquisition. Regardless, these data are consistent with our bioinformatic analyses, which indicates that both spacer acquisition and spacer loss contribute to type I-F CRISPR array diversity in *L. pneumophila* isolates (Figure 2).

**Figure 5:**
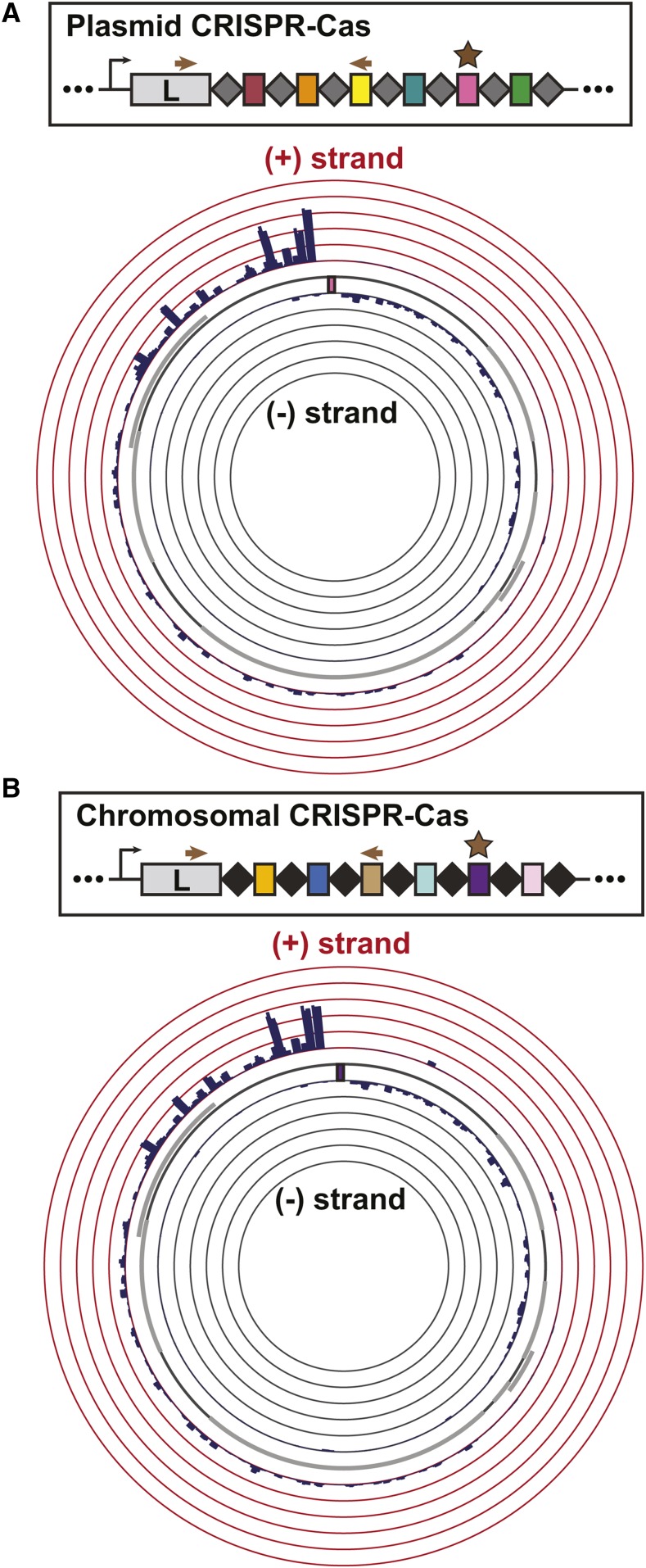
Characterization of self-primed spacer acquisition in the two Lens CRISPR-Cas systems. Bacterial transformants with targeted plasmids were passaged for 20 generations without antibiotic selection to enrich for spacer acquisition; the leader end of the CRISPR array was amplified and the amplicons were Illumina sequenced. Newly targeted protospacers were obtained from the raw reads using an in-house bioinformatics pipeline and visualized with Circos. The arrows in the simplified array schematic show the primer location for PCR amplification prior to sequencing, while the star denotes the priming spacer. The priming protospacer sequence is indicated by a colored box in the Circos plot. All data are the average of three biological replicates. A) The distribution of newly targeted protospacers mapped to the priming plasmid on the Circos plot reveals a strand bias in self-primed spacer acquisition within the Lens (pLPL) CRISPR-Cas system. The height of the bars indicates the number of spacers mapped to the position on the plasmid, up to 5% of total acquired spacers. B) The distribution of newly targeted protospacers mapped to the priming plasmid shows a similar pattern of self-primed spacer acquisition within the Lens (chromosome) CRISPR-Cas system to that of its pLPL CRISPR-Cas counterpart. Labeling as in (A).

Our observation that spacers downstream of spacer 1 in each type I-F system provided relatively modest protection led us to ask whether these spacers could nevertheless drive primed acquisition of new, more protective spacer sequences. To characterize the patterns of targeted spacer acquisition in the chromosomal and pLPL CRISPR-Cas systems, we amplified the leader-proximal region of each CRISPR array from wild-type populations that had been transformed with the plasmids targeted by their relatively permissive spacers (chromosomal: spacer 23; pLPL: spacer 50). We Illumina sequenced these PCR products and used an established bioinformatics pipeline (Rao *et al.* 2017) to identify newly acquired spacer sequences within each read (Table 1). We then mapped the target of each new spacer to the priming plasmid (Krzywinski *et al.* 2009) (Figure 5).

**Table 1:** The number of acquired spacers for the Lens (pLPL) and Lens (chromosome) CRISPR-Cas systems and their respective targets during self-priming

System	Number of acquired spacers*^a^*	% map to priming plasmid*^a^*	% map to Lens (chromosome)*^a^*	% map to Lens (pLPL)*^a^*	% unknown*^a^*
Lens (pLPL)	78,753	98.38	0.05	0.01	1.57
Lens (chromsosome)	86,789	98.66	0.01	0.00	1.33

a Data are the average of 3 biological replicates.

Both of the Lens type I-F CRISPR-Cas systems exhibited a biased distribution of acquired spacers (Figure 5, S1) consistent with what has been seen previously for the type I-F systems of *Pectobacterium atrosepticum* (Richter *et al.* 2014; Staals *et al.* 2016) and *Pseudomonas aeruginosa* (Vorontsova *et al.* 2015; Heussler *et al.* 2016). The majority of the newly targeted protospacers clustered around the priming sequence on the targeted plasmid, with the non-primed strand of DNA (the plus (+) strand) containing ∼75% of these protospacers (Figure 5). Consistent with observations in other type I-F systems (Richter *et al.* 2014; Staals *et al.* 2016; Heussler *et al.* 2016), switching the target sequence to the opposite strand led to an acquisition pattern that mirrored the original distribution observed when the (-) strand contained the targeted protospacer (Fig. S2). The spacer length distribution and PAM usage were also consistent with previous observations of other type I-F systems (Mojica *et al.* 2009; Cady *et al.* 2012; Richter *et al.* 2014; Vorontsova *et al.* 2015; Staals *et al.* 2016) (Fig. S2B, C, S3, S4). Taken together, these data suggest that spacer acquisition is qualitatively similar between the chromosomal and pLPL CRISPR-Cas systems.

### Permissive targeting by one system can lead to primed acquisition in the other system

**Figure 6:**
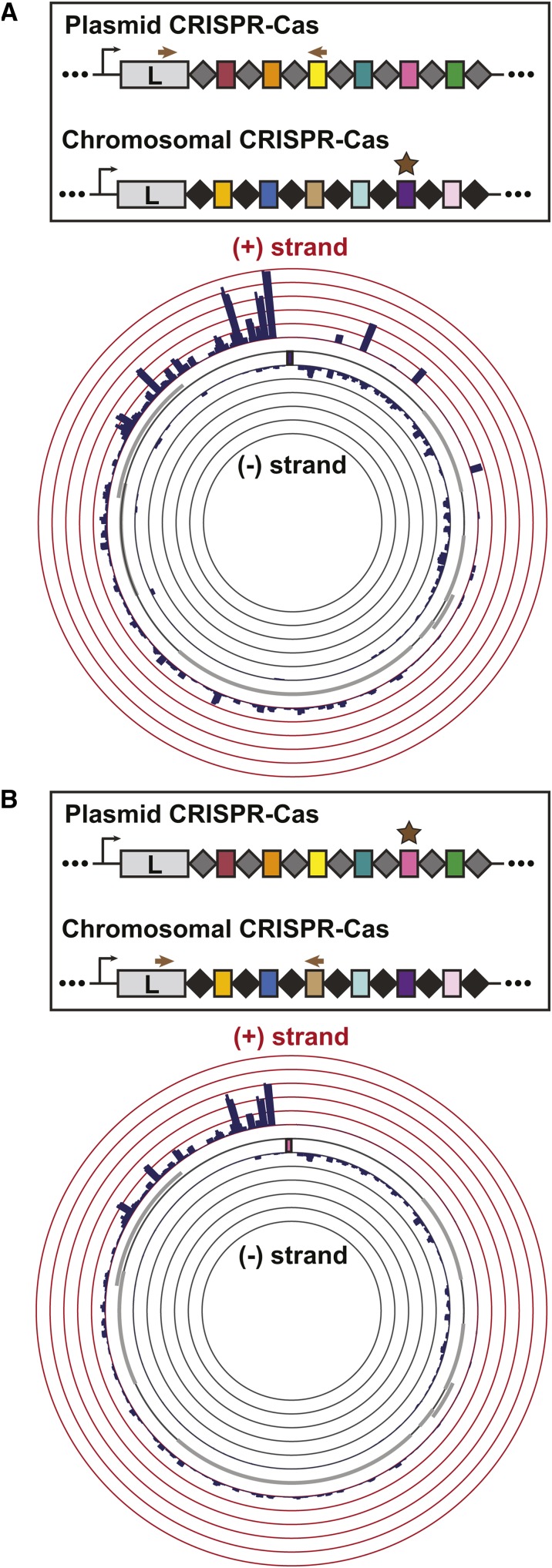
Characterization of cross-primed spacer acquisition in the two Lens CRISPR-Cas systems. The experimental set-up is the same as described in Figure 5. All data are the average of three biological replicates. The distribution of newly targeted protospacers mapped to the priming plasmid reveals cross-priming between the Lens (chromosome) and Lens (pLPL) CRISPR-Cas systems. (A) shows Lens (chromosome) primed, Lens (pLPL) array examined while (B) shows Lens (pLPL) primed, Lens (chromosome) array examined.

Since the chromosomal and pLPL CRISPR-Cas systems function in a very similar manner during targeted acquisition and share a high degree of homology within their *cas* genes and repeat sequences, we hypothesized that the priming of one system might lead to spacer acquisition in the other. Specifically, we tested whether introducing a protospacer-containing plasmid targeted by one CRISPR-Cas system would initiate a primed acquisition response in the second system. Indeed, this led to efficient spacer acquisition on the second array (Figure 6), with patterns largely indistinguishable from what we previously observed on the cognate array (Figure 5).

### Primed repopulation of collapsed arrays

**Figure 7:**
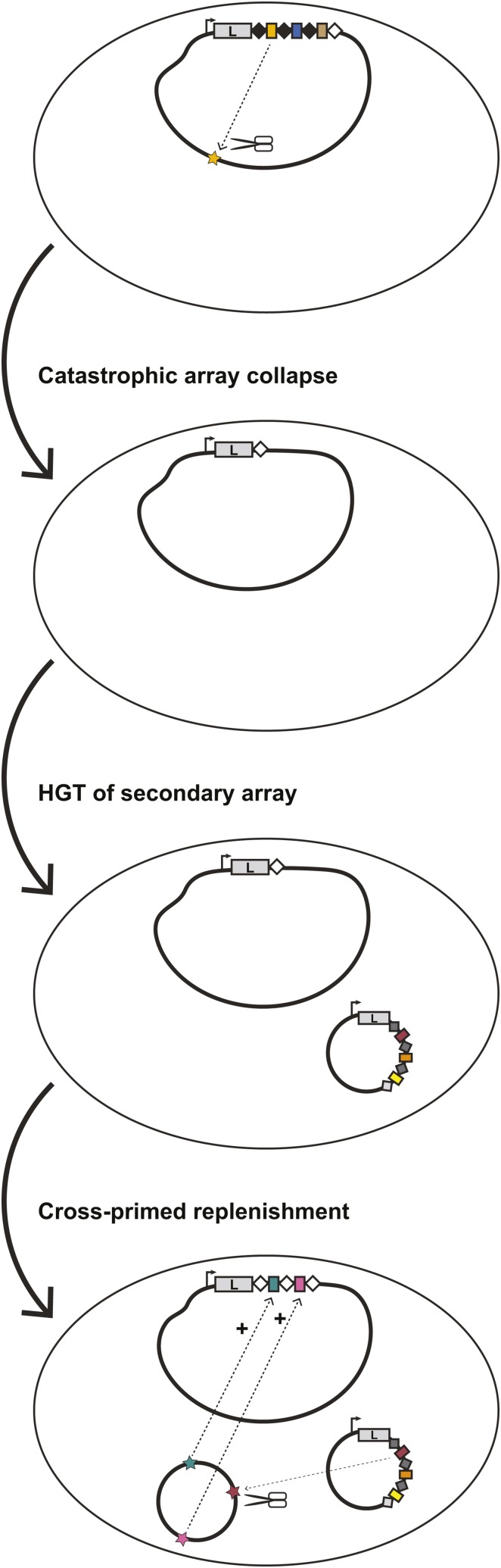
Cross-priming between two similar CRISPR-Cas systems can repopulate a collapsed CRISPR array. A bacterium with a CRISPR-Cas system could undergo a mass spacer deletion event through homologous recombination between the first (black) and last (white) repeat sequences. The remaining locus carries a single repeat (white). While such “catastrophic collapse” events are likely to occur randomly at a certain rate, a driver of such a collapse could be the acquisition of a self-targeting spacer (yellow), selecting for spacer loss. Horizontal acquisition of a second CRISPR-Cas array (*e.g.*, on a plasmid) is a first step toward replenishing the primary array. If cross-priming can occur between this secondary array and the collapsed array, the original CRISPR array is replenished, but bears an observable molecular scar - conversion of all the repeats to the sequence of the last repeat (white).

Based on our observations suggesting widespread horizontal inheritance of *L. pneumophila* type I-F systems (Figure 1), the diversity of spacer sequences (Figure 2), and the ability of closely related systems to prime each other (Figure 6), we next asked whether coincident CRISPR-Cas might provide a mechanism for replenishing collapsed chromosomal arrays. Spacer loss is one of several outcomes when the targeting of a particular sequence becomes detrimental to bacterial survival. In the lab, this occurs when we artificially “force” the coexistence of an efficiently targeted plasmid and an active CRISPR-Cas system through selection (Jiang *et al.* 2013) (Figure 4). Similar events are also likely to occur randomly or when CRISPR-Cas systems acquire self-targeting spacers at a low, but detectable rate (Figure 7) (Yosef *et al.* 2012; Datsenko *et al.* 2012; Savitskaya *et al.* 2013; Vorontsova *et al.* 2015; Staals *et al.* 2016; Rao *et al.* 2017).

In the most extreme instance, an entire array of spacers could be lost through recombination between the first and last repeats. Normally, such a loss could only be reversed by the relatively inefficient mechanism of naïve spacer acquisition (Yosef *et al.* 2012; Datsenko *et al.* 2012; Savitskaya *et al.* 2013). However, if such a collapsed array could be restored through primed acquisition driven by a coincident system, strains with multiple arrays would be inherently more resistant to the loss of CRISPR-Cas protection. Leveraging the two experimentally tractable type I-F systems of *L. pneumophila* str. Lens, we sought to bioinformatically and experimentally test some of these predictions.

**Figure 8:**
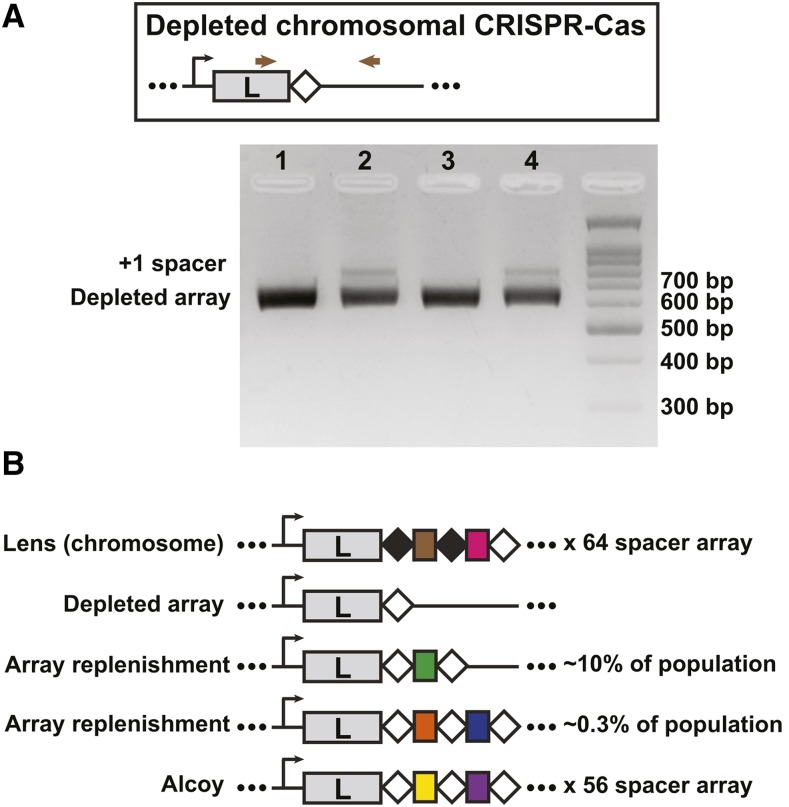
An experimentally depleted chromosomal CRISPR array can be replenished through the activity of a plasmid-based array. A) Replenishment of a depleted array in the lab. Allelic replacement was used to remove the entire array from the chromosomal Lens I-F system, leaving behind a single, last repeat sequence (see materials and methods). This strain was then transformed with a plasmid targeted by the pLPL (Lens plasmid-based) I-F array previously shown to drive primed acquisition (pLPL protospacer 50; see Figure 4). Spacer acquisition by the empty chromosomal array was analyzed using a PCR based screen where the leader-end of the CRISPR array was amplified and visualized on an agarose gel. Products from the transformed samples (samples 2 and 4) were compared to untransformed controls (samples 1 and 3). Samples 1 and 2 are from depleted array clone #1 and samples 3 and 4 are from depleted array clone #2. B) Repeat signatures of depletion/replenishment. The repeat structure of the experimentally replenished Lens CRISPR arrays resembles that of *L. pneumophila* str. Alcoy, suggesting a similar array depletion/replenishment event may have occurred within the Alcoy lineage. The frequency of acquiring one new spacer *vs.* two new spacers following replenishment in the Lens array depletion isolates was determined by Illumina sequencing.

First, we used allelic replacement to generate an *L. pneumophila* Lens strain in which the entire chromosomal array was replaced by a single copy of its last repeat, mimicking what would occur after complete spacer loss. Next, we transformed two independently derived array deletion strains with the pLPL type I-F priming plasmid (pLPL protospacer 50 plasmid) as above. Using PCR and Illumina sequencing, we observed robust spacer acquisition in the formerly depleted chromosomal CRISPR array (Figure 8, Table S4), indicating that primed acquisition can replenish the completely collapsed array of a coincidental type I-F system. As expected, the consensus repeat sequence of this replenished array adopted the same alternate sequence as the last repeat of the array.

We next examined the CRISPR (repeat) sequences in each of the isolates used in our earlier bioinformatic analyses for similar evidence of complete collapse followed by replenishment. In many of the arrays, there is a consensus repeat that is found throughout the majority of the array, with the last repeat in the array carrying a mutation (Table 2). This has been observed before in other systems (Jansen *et al*. 2002a; 2002b; Horvath *et al.* 2008; Lopez-Sanchez *et al.* 2012). We have previously shown that for the *L. pneumophila* I-C system, complete loss of an array leads overwhelmingly to a recombination product that resembles the alternate last repeat sequence (Rao *et al.* 2017). This is presumably because recombination is favored to occur between the region of identity that falls upstream of the mutated nucleotides of the last repeat. A similar sequence structure exists in the type I-F repeats, predicting a similar outcome after complete array loss. In *L. pneumophila* isolates Alcoy, JFIM01, LBAN01 and LBAV01, their consensus repeat (found throughout the CRISPR array) is identical to their last repeat. Intriguingly, the sequence that remains is identical to the last repeat found in other type I-F isolates – what one would predict if they were the product of complete array collapse followed by subsequent replenishment (Figure 8).

**Table 2:** **The repeat sequences of L. pneumophila type I-F CRISPR-Cas systems***^a^*

Strain	# of spacers in the array	Consensus repeat (5′-3′)*^b^*	Mutated last repeat (5′-3′)*^b^*
Lens (chromosome)	64	GTTCACTGCCGCACAGGCAGCTTAGAAA	GTTCACTGCCGCACAGGCAGCTTAGAA**G**
FFI104	55	GTTCACTGCCGCACAGGCAGCTTAGAAA	GTTCACTGCCGCACAGGCAGCTTAGAA**G**
Alcoy	56	GTTCACTGCCGCACAGGCAGCTTAGAA**G**	–
Lens (pLPL)	53	GTTCACTGCCG**T**ACAGGCAGCTTAGAAA	GTTCACTGCCG**T**ACAGGCAGCTTAGAA**G**
Missisauga-2006 (plasmid)	74	GTTCACTGCCG**T**ACAGGCAGCTTAGAAA	GTTCACTGCCG**T**ACAGGCAGCTTAGAA**G**
C8_S (plasmid)	24	GTTCACTGCCG**T**ACAGGCAGCTTAGAAA	GTTCACTGCCG**T**ACAGGCAGCTTAGAA**G**
Calgary2017	8	GTTCACTGCCG**T**ACAGGCAGCTTAGAAA	GTTCACTGCCG**T**ACAGGCAGCTTAGAA**G**
FJAB01	59	GTTCACTGCCGCACAGGCAGCTTAGAAA	–*^c^*
FJAD01	48	GTTCACTGCCG**T**ACAGGCAGCTTAGAAA	GTTCACTGCCG**T**ACAGGCAGCTTAGAA**G**
FJAJ01	55	GTTCACTGCCG**T**ACAGGCAGCTTAGAAA	GTTCACTGCCG**T**ACAGGCAGCTTAGAA**G**
FJAM01	72	GTTCACTGCCG**T**ACAGGCAGCTTAGAAA	GTTCACTGCCG**T**ACAGGCAGCTTAGAA**G**
FJBW01	69	GTTCACTGCCG**T**ACAGGCAGCTTAGAAA	GTTCACTGCCG**T**ACAGGCAGCTTAGAA**G**
FJDP01	55	GTTCACTGCCG**T**ACAGGCAGCTTAGAAA	GTTCACTGCCG**T**ACAGGCAGCTTAGAA**G**
FJEJ01	56	GTTCACTGCCGCACAGGCAGCTTAGAAA	–*^c^*
FJMD01	47	GTTCACTGCCG**T**ACAGGCAGCTTAGAAA	GTTCACTGCCG**T**ACAGGCAGCTTAGAA**G**
JFID01	92	GTTCACTGCCGCACAGGCAGCTTAGAAA	GTTCACTGCCGCATAGGCAG**TGGGTTG**
JFIM01	39	GTTCACTGCCGCACAGGCAGCTTAGAA**G**	–
LBAK01	53	GTTCACTGCCG**T**ACAGGCAGCTTAGAAA	GTTCACTGCCG**T**ACAGGCAGCTTAGAA**G**
LBAN01	54	GTTCACTGCCGCACAGGCAGCTTAGAA**G**	–
LBAV01	34	GTTCACTGCCGCACAGGCAGCTTAGAA**G**	–
UGOL01	129	GTTCACTGCCGCACAGGCAGCTTAGAAA	GTTCACTGCCGCACAGGCAGCTTAGAA**G**
UGOM01	53	GTTCACTGCCGCACAGGCAGCTTAGAAA	GTTCACTGCCGCACAGGCAGCTTAGAA**G**

a The isolates listed in this table reflect those present in the phylogeny from figure 1B.

b Mutations in the last repeat relative to the consensus repeat, and in the two primary consensus repeats relative to each other, are bolded.

c While these strains do not possess a mutated last repeat, their 3^rd^ last repeat is mutated relative to the consensus repeat.

## Discussion

Horizontal gene transfer is a driving force in shaping bacterial biology and pathogenicity (Ochman *et al.* 2000; Juhas 2013; Hall *et al.* 2017). With respect to *L. pneumophila*, comparative genomic studies have highlighted the importance of horizontal gene transfer, mobile genetic elements, and homologous recombination in shaping the bacterium’s evolutionary trajectory (Cazalet *et al.* 2004; 2008; D’Auria *et al.* 2010; Gomez-Valero *et al.* 2011; Sánchez-Busó *et al.* 2014; Gomez-Valero *et al.* 2014; McAdam *et al.* 2014; Burstein *et al.* 2016; David *et al.* 2017). This aspect of *L. pneumophila* biology extends to the presence and maintenance of plasmid-based CRISPR-Cas systems (D’Auria *et al.* 2010; Gomez-Valero *et al.* 2011; Rao *et al.* 2016). Our bioinformatic analyses suggest that type I-F CRISPR-Cas systems are horizontally distributed in this species (Figure 1). These CRISPR arrays have also undergone extensive spacer acquisition and some spacer loss (Figure 2). While only three of the type I-F CRISPR-Cas systems we describe are present on annotated plasmids (str. Lens, str. Mississauga-2006 and str. C8_S, Figure 2), this is likely an underestimation due to the nature of analyzing draft genomes. Notably, an additional six strains (FJAD01, FJAJ01, FJAM01, FJBU01, FJBW01 and FJMD01, Figure 2) have systems that reside on contigs with overlapping sequence (78-89 nt) on either end, suggesting that they may also reside on plasmids or other mobile elements. Indeed, horizontal acquisition of type I-F systems is well-established in other species. For instance, in *Vibrio* species, 97% of identified type I-F systems are encoded on mobile genetic elements (McDonald *et al.* 2019). Like *L. pneumophila*, it’s hypothesized these systems have been acquired by their host through horizontal gene transfer (McDonald *et al.* 2019). Our data, however, suggest that such transfer may not be merely a mechanism by which isolates acquire CRISPR-Cas protection, but could also maintain existing defensive capabilities through the unique spacer dynamics provided by two inter-priming arrays. These inter-priming arrays can be part of two different systems, as demonstrated by our data, but could also occur in a system where two (or more) different arrays share a set of *cas* genes (Swarts *et al.* 2012; Datsenko *et al.* 2012; Staals *et al.* 2013; 2014; Majumdar *et al.* 2015; Elmore *et al.* 2015; Silas *et al.* 2017). For example, in *E. coli* str. K12, it has been shown that two different CRISPR arrays can be populated by the same set of *cas* genes (Swarts *et al.* 2012; Datsenko *et al.* 2012).

We propose that when a bacterium acquires a second, closely related CRISPR-Cas system, it gains a mechanism by which depleted CRISPR arrays can be repopulated (Figure 7). We have modeled such an event in the lab, made predictions about what the signatures of such events would be, and provide genomic data to suggest it may have occurred on several occasions within our collection of sequenced isolates. One obvious line of future investigation would be to observe whether such patterns are present in other species with mobilized type I CRISPR-Cas and perhaps absent in instances where CRISPR-Cas is acquired primarily through vertical inheritance. Such signatures may be a way to detect these events even if they are rare or transient. Notably, we have observed only one strain with two type I-F CRISPR-Cas systems (*L. pneumophila* str. Lens). Two additional strains (*L. pneumophila* str. Mississauga-2006 and FJAD01) possesses a type I-C system on its chromosome and a type I-F system on a plasmid. These data suggest that *stable* co-occurrence of CRISPR-Cas systems appears to be rare in the *L. pneumophila* isolates described so far.

Lastly, while we think of spacer loss as a predominantly negative event (loss of protection), it is likely to play a more nuanced role in the maintenance of CRISPR-Cas activity. Jiang and colleagues have presented work that demonstrated loss of CRISPR-Cas to maintain beneficial plasmids and discussed the trade-offs of CRISPR immunity *vs.* beneficial genetic elements (*i.e.*: confers resistance, etc.) (Jiang *et al.* 2013). Using a conjugation assay with a targeted resistance plasmid, they observed loss of the spacer targeting the plasmid (13% of the transconjugants), mutations in CRISPR-Cas that abolished its function (37% of the transconjugants) or partial/complete deletion of the CRISPR-Cas locus (50% of the transconjugants) (Jiang *et al.* 2013). Their competition data suggested that even with loss of the entire system, there was little to no fitness cost associated with this loss (Jiang *et al.* 2013).

Array length is the product of a dynamic process whose impact on adaptation, expression, and interference remains largely unexplored. Many of the type I-F systems in *L. pneumophila* have different array lengths, ranging from 8 spacers to 129 spacers, with an average length of 61 spacers (Table 2). A global analysis of class I CRISPR arrays found that the average array length for type I-F systems was 33 spacers, with statistically significant differences between the array lengths of different type I subtypes (Toms and Barrangou 2017). Accordingly, if spacer acquisition is a driving force in array divergence, it is likely coupled to spacer loss. Close examination of the mechanisms driving spacer loss in these systems - and their subsequent impact on CRISPR-Cas functionality - will be crucial to further testing the model of array diversification in *L. pneumophila*.
